# 569. Utility of Repeat Molecular Diagnostic Testing in the Laboratory Diagnosis of Pulmonary Tuberculosis

**DOI:** 10.1093/ofid/ofad500.638

**Published:** 2023-11-27

**Authors:** Jonathan Chia

**Affiliations:** Tan Tock Seng Hospital, Singapore, Singapore, Singapore

## Abstract

**Background:**

Molecular diagnostic methods are commonly used to supplement microscopy and culture in the rapid diagnosis of pulmonary tuberculosis (TB). While international guidelines recommend the collection of multiple sputum samples for microscopy and culture due to their low sensitivity, the utility of performing multiple molecular tests is less well-defined. A retrospective analysis of molecular diagnostic testing in a high pre-test probability population was conducted to determine the impact of repeated testing on achieving diagnosis.

**Methods:**

A review of 10406 requests for *Mycobacterium tuberculosis* polymerase chain reaction (PCR) testing, originating from the TB contact screening and treatment clinic of a national Infectious Diseases specialist centre, was conducted for the period of 1 Jan - 31 Dec 2022. 5336 patients with valid test results were identified. (Table 1)

PCR testing was performed using the Xpert® MTB/RIF (Cepheid) PCR assay, either in the clinic as a point-of-care test, or in the hospital’s microbiology laboratory. Acid-fast microscopy was performed in the microbiology laboratory using a combination of fluorochrome and Ziehl-Neelsen staining, while cultures were referred to a Biosafety Level 3 laboratory at another institution for culture and susceptibility testing using the Mycobacteria Growth Indicator Tube (MGIT) system.

**Table 1:**
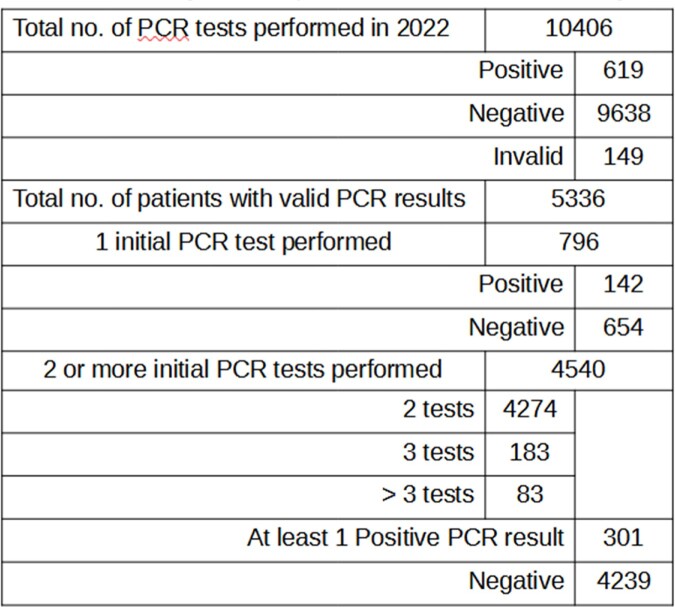
PCR testing ordered by a tuberculosis contact screening and treatment clinic in 2022

**Results:**

Out of 5336 patients identified, 4540 (85.0%) had 2 or more PCR tests performed within the first month of their initial encounter.

443 patients (8.3%) tested PCR-positive in total. 385 tested positive on their first test; further tests after the first identified another 58 cases. Compared to cumulative culture results, the sensitivity and specificity of the first PCR test performed in the target population was 65.2% and 98.3% respectively, with a negative predictive value (NPV) of 96.8%. Repeat PCR testing increased sensitivity to 69.3% and NPV to 97.5%. (Tables 2 and 3) 74 initially-negative patients would need to be retested to identify an additional PCR-positive case, with potential costs of SGD $14,504 per case ($196 per test).

**Table 2:**
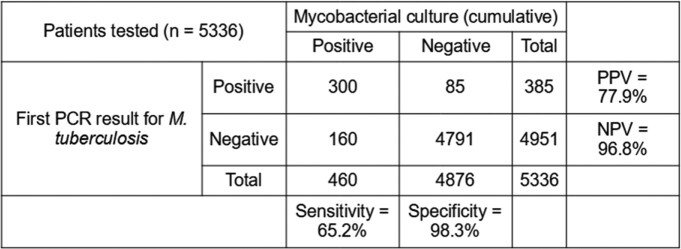
Comparison of single PCR test performance against cumulative mycobacterial culture PPV = Positive Predictive Value NPV = Negative Predictive Value

**Table 3:**
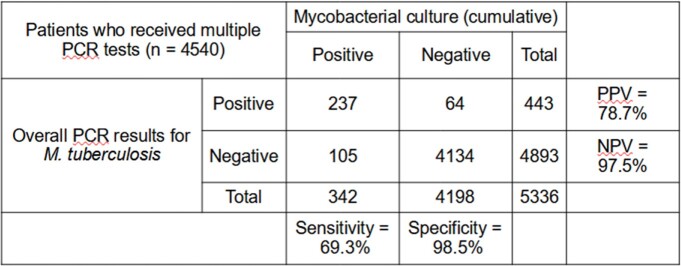
Comparison of repeated PCR test performance against cumulative mycobacterial culture PPV = Positive Predictive Value NPV = Negative Predictive Value

**Conclusion:**

For purposes of resource allocation, rationalisation of repeat testing in the diagnosis of pulmonary TB should be considered as an alternative to routine retesting, even in a high-risk population.

**Disclosures:**

**All Authors**: No reported disclosures

